# Closing the Gap in VTE Prophylaxis: The Role of Clinical Decision Support∗

**DOI:** 10.1016/j.jacadv.2023.100601

**Published:** 2023-09-01

**Authors:** Mark Sonderman, Quinn S. Wells

**Affiliations:** a Division of Cardiology, Department of Medicine, University of Washington Medical Center, Seattle, Washington, USA; b Division of Cardiovascular Medicine, Department of Medicine, Vanderbilt University Medical Center, Nashville, Tennessee, USA

**Keywords:** clinical decision support, thromboprophylaxis, venous thromboembolism

**Figure:**
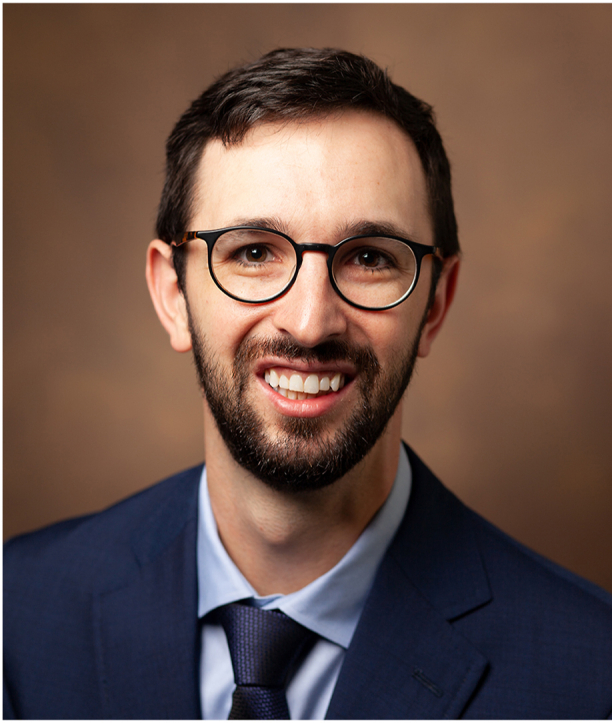

Hospital-associated venous thromboembolism (VTE) represents a significant and preventable cause of morbidity and mortality, with nearly half of all VTE deaths in the United States each year occurring during or shortly after hospitalization.^1^ VTE is the most common cause of preventable death in medical inpatients, surpassing nosocomial pneumonia, catheter-associated bloodstream infections, and adverse drug events.^2^^,^^3^ Although chemical thromboprophylaxis reduces the risk of VTE by approximately 30%, less than one-third of medical inpatients receive indicated prophylaxis.^4^, ^5^, ^6^

Clinical decision support (CDS) encompasses computational tools that provide patient data and guidelines to health care providers within their clinical workflow, aiming to increase the practice of evidence-based medicine. One promising application of CDS is the use of electronic health record (EHR) alerts to promote VTE prophylaxis prescribing. A meta-analysis of CDS tools revealed increased appropriate thromboprophylaxis use and a 36% reduction in the rate of symptomatic VTE at 3 months.^7^ While similar tools for postdischarge thromboprophylaxis have shown increased anticoagulant use, they have not demonstrated a similar reduction in VTE events.^8^ Notably, alert-based CDS tools at the time of prescription have proven most effective at increasing thromboprophylaxis prescribing rates, surpassing educational sessions or auditing, and feedback interventions.^7^^,^^9^

The paper by Spyropolous et al^10^ in this issue of *JACC: Advances*, present the results of a cluster randomized trial of an integrated CDS tool for chemical thromboprophylaxis in medical inpatients. Across 4 hospitals (2 clusters, each with 2 hospitals) with over 10,000 patients, they found that hospitals in the intervention cluster had a significantly increased rate of appropriate thromboprophylaxis use, both during hospitalization and at discharge. Fewer thromboembolic events were observed in the intervention group without any increase in major bleeding. The observed decrease in VTE events (2.7% vs 3.3%) aligns with similar reductions seen in other CDS alert-based trials.^7^

Several aspects of the experimental intervention are commendable and demonstrate important CDS principles. For example, their CDS tool, at least in theory, was interoperable, with the goal of supporting integration into any EHR. Considering that no single EHR vendor holds more than 37% of the market share and that rural and critical access hospitals are more likely to employ different EHR platforms, the ability of CDS tools to work irrespective of EHR vendor is an important goal for the CDS field.^11^ Additionally, the authors prepopulated data whenever possible, minimizing the effort required from health care providers to utilize the CDS tool. This likely contributed to the remarkable rate of VTE prophylaxis use among eligible patients. Lastly, placing the CDS tool at critical clinical decision-making points (admission orders, history and physical documentation, and discharge order reconciliation) increased the probability that that tool was readily available to clinicians at relevant points in clinical workflow without overwhelming them with excessive alerts.^12^^,^^13^

Despite thoughtful implementation and encouraging findings, there are some features of the study that should be considered when evaluating the representativeness and generalizability of results. One important, unforeseeable factor was the COVID-19 (SARS-CoV-2) pandemic. The selection of study hospitals was based on historical medical admission data and assumed similar subject groups. However, investigators could not have anticipated that over 20% of all subjects in the study would be hospitalized with a novel infection. As the authors point out, differences in rates of COVID-19 inpatients (25.8% in the intervention group vs 20.1% in the control) may have contributed to the significant increase in mortality in the intervention group (9.1% vs 7.0%). Given that the intervention reduced the rate of thromboembolic events and rates of major bleeding were low, the difference in mortality suggests that these populations were not similar. I applaud the authors for evaluating their intervention based on outcomes rather than process changes, as is often seen with CDS studies.^14^ Nonetheless, the significant difference in mortality must be considered when interpreting their finding that the tool reduced the rate of thromboembolic events.

Another limitation in the generalizability of the results is that the reported tool adoption rate of 77.8% does not fully reflect the actual use among eligible patients. Only 5,249 out of 8,743 medical patients (60.0%) admitted to the intervention hospital who were not receiving therapeutic anticoagulation underwent evaluation by the tool. Several factors contributed to this, including provider opt-outs, instances where VTE prophylaxis was not evaluated during hospitalization, and the exclusion of patients with a history of atrial fibrillation not on anticoagulation. Therefore, while the promising rate of appropriate thromboprophylaxis use among patients who underwent evaluation by the tool is noteworthy (80%), an honest assessment of this tool’s impact requires pragmatic data on how its implementation affects all medical inpatients eligible for VTE prophylaxis, even in cases where the tool is not used.

As CDS tools gain prominence in clinical practice, assessing their clinical impact relative to costs becomes crucial. Unlike drug trials, which focus primarily on adverse health effects for study subjects, evaluating CDS tools requires consideration of how these tools integrate into clinicians' workflow.^15^ Several key principles guide the implementation of CDS tools. First, CDS should only be introduced if desired by the intended recipients. If users perceive no value in the CDS alert, its presence may be counterproductive, offering no benefit and reducing perceived value of other CDS tools. Second, integration into workflow should be carried out in the least disruptive manner possible. Third, evaluation should consider alert fatigue and workflow changes for an objective assessment of benefit vs cost. Lastly, involving practicing clinicians in the implementation process is essential for anticipating tool reception and usage.^14^

In conclusion, Spyropolous et al^10^ have developed a CDS tool that, when combined with clinician education, addresses a shortcoming in the care of acutely ill medical inpatients. High provider prescribing rates for VTE prophylaxis were observed after education, with additional improvements in both VTE prophylaxis use and VTE occurrence upon adding a clinical alert. However, limitations include the nonsimilar patient populations and the tool’s nonuse in a significant portion of medical inpatients. The observed reduction in VTE events, similar to prior CDS tools, further strengthens the evidence that implementing most alert-based tools will decrease the incidence of hospital-associated VTE. The authors’ contributions to the field are notable as they addressed interoperability concerns and emphasized hard outcomes (all-cause mortality, total thromboembolic events) over process improvements. Further steps should involve assessing the durability of these results over time and evaluating the interoperability of diverse hospital systems.

## Funding support and author disclosures

The authors have reported that they have no relationships relevant to the contents of this paper to disclose.
